# Metastatic Pancreatic Adenocarcinoma to Umbilical Skin

**DOI:** 10.7759/cureus.24568

**Published:** 2022-04-28

**Authors:** Maryam Aghighi, Mohammad Bagher Shokravi, Maral Rahvar

**Affiliations:** 1 Pathology, Harbor University of California Los Angeles Medical Center, Torrance, USA; 2 Pathology, M.B. Shokravi Office, North Vancouver, CAN; 3 Pathology and Laboratory Medicine, Lions Gate Hospital, Vancouver, CAN

**Keywords:** metastatic tumor, immunohistochemistry staining, umbilical skin, pancreatic adenocarcinoma, sister mary-joseph nodule

## Abstract

Sister Mary Joseph nodule (SMJN) is a rare metastasis to umbilical skin originating from internal tumors including the stomach, ovary and large intestine and less commonly from pancreatic cancers. We report an uncommon case of metastatic pancreatic adenocarcinoma to umbilical skin.

An 85-year-old female presented with a 1.8 cm protrusion of the right lateral umbilicus. The CT scan showed a 3.5 cm pancreatic lesion, peritoneal carcinomatosis and abdominal lymphadenopathy.

Histology examination revealed atypical infiltrative glandular structures. Immunohistochemistry showed positive CK7, negative CDX2 and P53 with mutated patterns. These were consistent with metastatic adenocarcinoma most consistent with pancreatobiliary or upper GI origin.

CK7 expresses in the ductal cells in pancreatic ductal adenocarcinoma. While CDX2 is positive in intestinal-type adenocarcinoma, it is negative in pancreatic ductal adenocarcinoma. The diagnosis of adenocarcinoma is rendered based on the presence of a pancreatic lesion in CT scan, positive CK7 and negative CDX2 in umbilical nodule tumor cells in the current patient.

## Introduction

Metastasis of internal tumors to umbilical skin, known as Sister Mary Joseph nodule (SMJN), is uncommon [1,2]. The metastasis to the umbilical skin is more common than other parts of the skin [3,4]. Sir Hamilton Bailey described SMJN in 1949 and there have been reports of more than 600 cases in the literature mainly from the stomach, ovary and large intestine since then [5]. Additionally, these nodules may be primarily associated with melanoma, squamous cell carcinoma or sarcoma [6]. The primary tumor in 15% of cases has not been found [7]. Metastatic disease from pancreatic cancers has been less than 10% of all the reported cases [7]. The main type of these tumors is adenocarcinoma [8]. Cholangiocarcinoma, anaplastic carcinoma and non-Hodgkin lymphoma are other histological types that have been described.

We report a rare case of metastatic pancreatic adenocarcinoma to umbilical skin in an elderly patient.

## Case presentation

**Figure 1:**
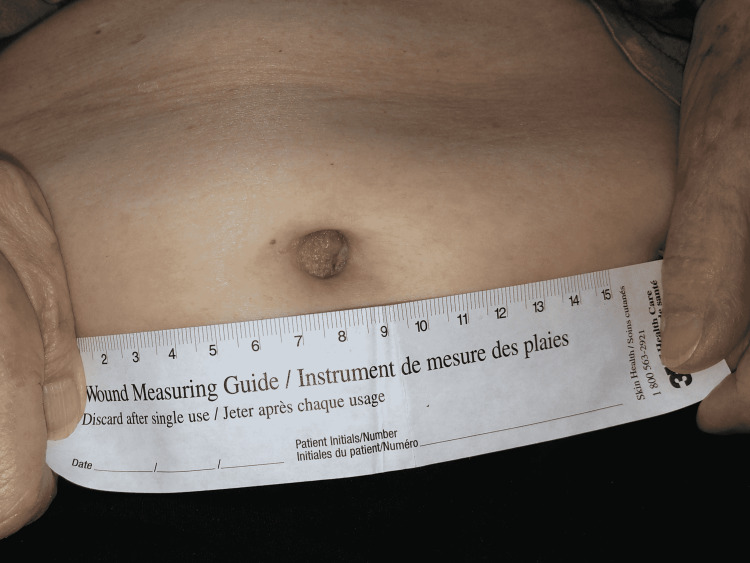
Clinical presentation of Nodule of Sister Mary-Joseph: 1.8 cm mass at the right lateral umbilicus

An 85-year-old female presented to the dermatology clinic via teledermatology program with soft tissue prominence of the right lateral umbilicus measuring 1.8 cm (Figure 1).

**Figure 2:**
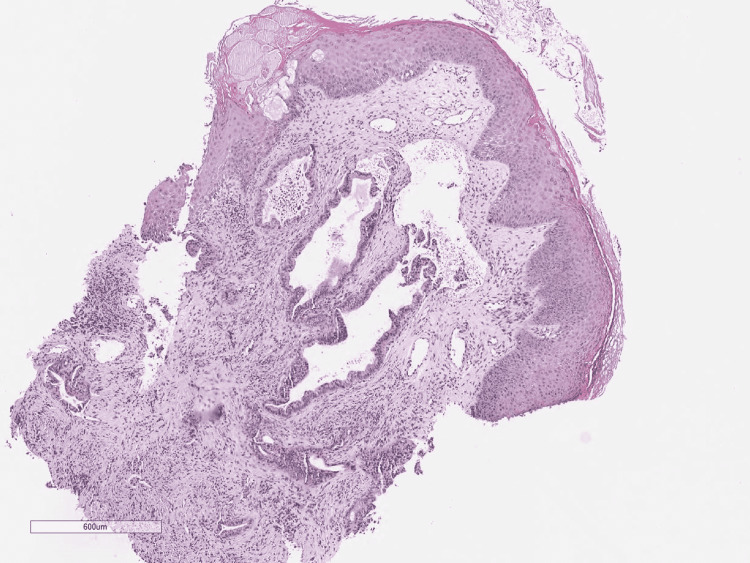
Metastatic pancreatic adenocarcinoma to skin: Atypical glands in the skin dermis, H&E (40x) H&E: Hematoxylin and Eosin stain

**Figure 3:**
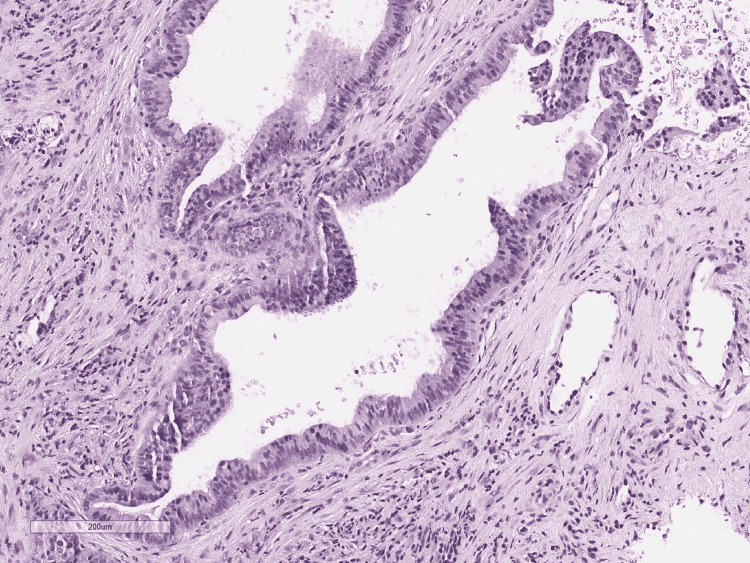
Metastatic pancreatic adenocarcinoma to skin: Infiltrating malignant glands in the skin dermis, H&E (100x) H&E: Hematoxylin and Eosin stain

A punch biopsy was obtained for the characterization of the lesion. Histologic examination showed atypical infiltrative glandular structures (Figure 2 and Figure 3).

**Figure 4:**
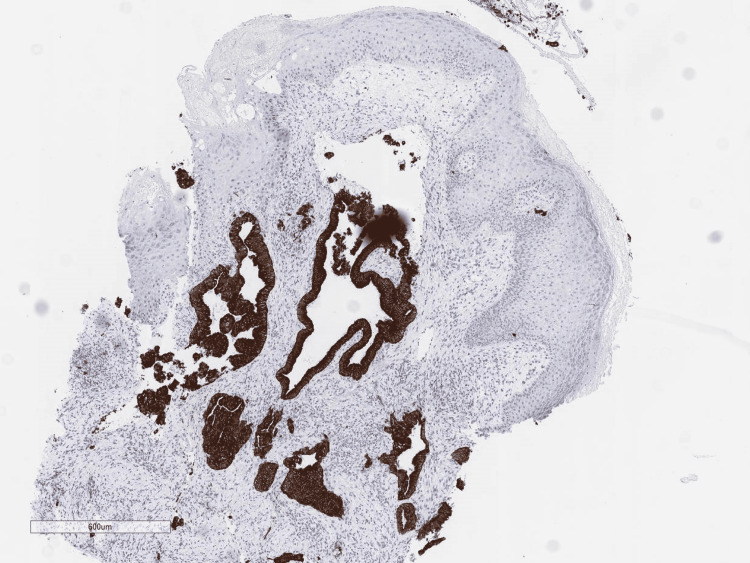
Positive CK7 stain: Membranous and cytoplasmic dark brown stain highlights the abnormal glands in the dermis (20x)

**Figure 5:**
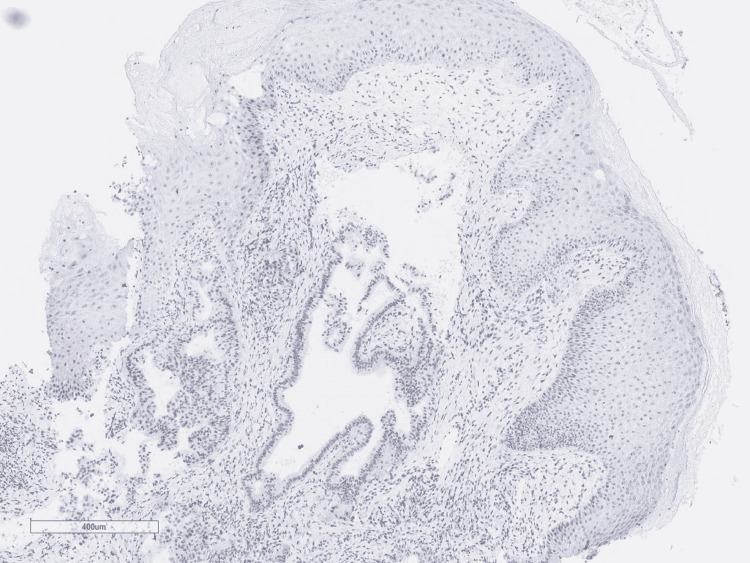
Negative CDX2 stain (40x)

By immunohistochemistry, these neoplastic cells were positive for CK7 (Figure 4) and negative for p63, TTF-1, CDX2 (Figure 5), estrogen receptor, PAX8, HER2 and GATA3 stains. Staining for p53 showed a mutated (overexpressed) pattern. Immunohistochemical stains for mismatch repair (MMR) proteins revealed a normal MMR profile. Mutated BRAFV600E protein was not identified. Overall, these findings were consistent with metastatic adenocarcinoma. Given the immunoprofile, intraabdominal origin such as pancreatobiliary or upper GI tract was favored.

**Figure 6:**
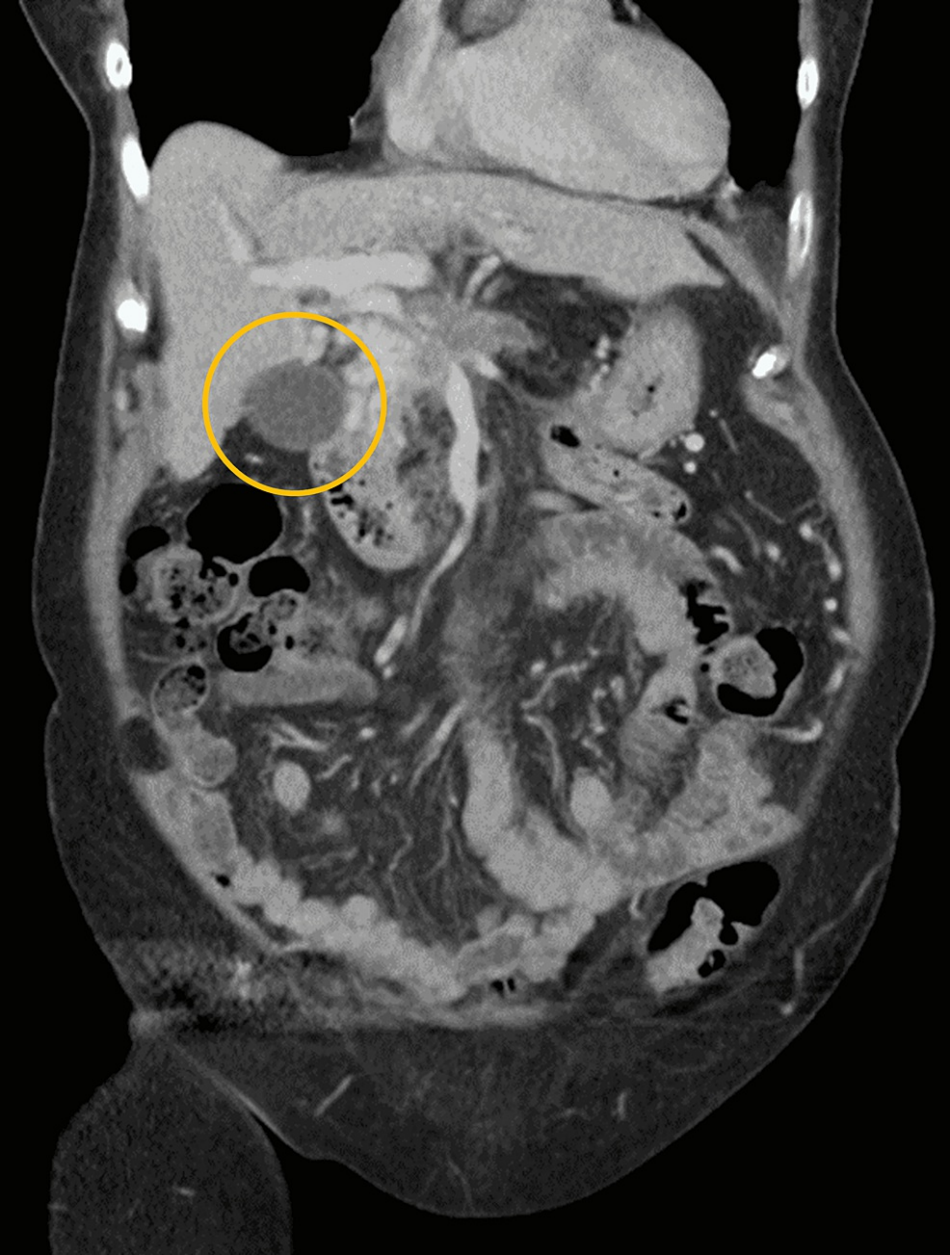
CT scan showing pancreatic head mass highlighted by the yellow circle

Further diagnostics workup was performed to find the primary origin of malignancy. A computerized tomography (CT) scan showed a 3.5 cm mass that diffusely involved the pancreatic head resulting in occlusion of the portal vein with cavernous transformation (Figure 6). The superior mesenteric vein was obstructed in the region as was the splenic vein.

After discussing the findings, the patient decided to decline the treatment and was referred to the palliative team for follow-up.

## Discussion

The exact pathophysiology of SMJN is unknown. It may be related to spreading through the peritoneum and pancreatic tumor cells implantation on the umbilicus or direct metastatic invasion from peritoneal tumors. There are other possible mechanisms of spreading tumor cells to the umbilicus including tumor cell invasion through lymphatic system, vessels or the umbilical ligament [9,10]. The most common cause of SMJN is metastasis from internal tumors including pancreatic malignancy. Pancreatic tumor is often diagnosed at a late stage after being asymptomatic for a long period. It has a poor prognosis and patients usually survive a few months. Imaging methods and tumor markers can be used for the diagnosis of the primary tumor. SMJN cytokeratin (CK) immunohistochemistry is helpful to find the primary tumors. Most pancreatic ductal adenocarcinoma cases express CK7 which highlights the ductal cells of the pancreas. While most pancreatic ductal adenocarcinomas do not express CDX2, intestinal-type adenocarcinoma is positive for CDX2. The elevated value of CA19-9 may be an indication of pancreatic cancer [11,12].

In the current case, there was a pancreatic head mass in CT scan, CK7 positive tumor cells in umbilical nodule with negative CDX2, and histopathology of the pancreatic tumor indicative of adenocarcinoma confirmed by biopsy.

Chemotherapy and radiotherapy have been used for the treatment of patients. In cases with SMJN metastasis alone, surgery and adjuvant therapy have been shown to improve patient survival.

## Conclusions

While CK7 is positive in pancreatic ductal adenocarcinoma, CDX2 is negative. CDX2 is positive in intestinal-type adenocarcinoma. Therefore, in the current case, the diagnosis of adenocarcinoma was rendered based on the pancreatic mass in the CT scan, CK7 positivity and CDX2 negativity in umbilical nodule tumor cells.
